# Association of 2184AG Polymorphism in the RAGE Gene with Diabetic Nephropathy in Chinese Patients with Type 2 Diabetes

**DOI:** 10.1155/2015/310237

**Published:** 2015-12-03

**Authors:** Wei Cai, Jian Li, Ji-Xiong Xu, Ying Liu, Wei Zhang, Jun-Ren Xiao, Ling-Yan Zhu, Jian-Ying Liu

**Affiliations:** ^1^Department of Endocrinology and Metabolism, First Affiliated Hospital of Nanchang University, Nanchang, Jiangxi 330006, China; ^2^Department of Medical Genetics and Cell Biology, Medical College of Nanchang University, Nanchang, Jiangxi 330006, China; ^3^Department of Endocrinology and Metabolism, First People's Hospital of Xinxiang City, Xinxiang, Henan 453000, China; ^4^Department of Endocrinology and Metabolism, Third Affiliated Hospital of Ganzhou Medical College, Ganzhou, Jiangxi 341000, China

## Abstract

*Objective*. The interaction between advanced glycation end products and their cellular receptor (RAGE) has an important role in the pathogenesis of diabetic microvascular complications. The aim of this study was to investigate the relationship between the 2184A/G polymorphism in the RAGE gene and diabetic nephropathy in Chinese Han patients with type 2 diabetes mellitus. *Methods*. A total of 868 patients with type 2 diabetes mellitus (486 without and 382 with diabetic nephropathy) were enrolled in this study. The genotype and allele frequencies of the 2184A/G polymorphism were detected using the polymerase chain reaction-restriction fragment-length polymorphism method. *Results*. The G allele and AG + GG genotype frequencies in patients with diabetic nephropathy were significantly lower than those in patients without diabetic nephropathy (*P* = 0.001 and *P* = 0.005, resp.). After adjustments for possible confounders, multivariate logistic regression analyses showed that the 2184A/G polymorphism was independently associated with diabetic nephropathy (OR = 0.46, 95% CI: 0.22–0.92, *P* = 0.028). *Conclusions*. Our study indicated that the 2184A/G polymorphism in the RAGE gene was significantly associated with diabetic nephropathy in Chinese Han patients with type 2 diabetes.

## 1. Introduction

Diabetic nephropathy is one of the main microvascular complications of diabetes mellitus and is a leading cause of end-stage renal disease [1]. It is widely accepted that diabetic nephropathy is a heterogeneous disorder caused by the interaction between environmental and genetic factors [2]. Although poor glycemic control and diabetic duration are the major risk factors in the development of diabetic nephropathy, hypertension, hyperlipidemia, and genetic factors are also associated with its development. The molecular mechanisms that underlie the pathogenesis of diabetic nephropathy remain unclear.

Hyperglycemic conditions in diabetes lead to the formation and accumulation of advanced glycation end products (AGEs) in tissues. In diabetes, the kidney is an important site for AGE accumulation and was one of the first diabetic tissues in which AGE accumulation was observed [3]. It has been suggested that AGEs might play a role in the pathogenesis of diabetic nephropathy and the progression to renal failure [4]. The actions of AGEs are mainly dependent on their specific cell-surface receptor, the receptor for AGE (RAGE) [5]. With activation of signal transduction cascades and transcription factors such as nuclear factor- (NF-) *κ*B, the interaction between AGEs and RAGE induces oxidative stress and increased expression of inflammatory and prothrombotic species [6]. There is growing evidence to suggest that RAGE has an important role in diabetic vascular complications [7–9].

Genetic polymorphisms in the RAGE gene may alter its activity following AGE binding and, thereby, may influence the development of diabetic vascular complications. The 2184A/G polymorphism, which is located on intron 8 of the RAGE gene, has been reported to be associated with microvascular dermatoses and antioxidant status [10, 11]. In this study, we aimed at investigating the association of RAGE 2184A/G polymorphism with diabetic nephropathy in Chinese Han patients with type 2 diabetes.

## 2. Materials and Methods

### 2.1. Subjects

We selected subjects with type 2 diabetes (*n* = 868) of Chinese Han ethnicity who were inpatients at the Department of Endocrinology and Metabolism at the First Hospital of Nanchang University between June 2010 and December 2014. Type 2 diabetes was diagnosed according to the 2003 American Diabetes Association diagnostic criteria for diabetes, and subjects were divided into two groups: without diabetic nephropathy (*n* = 486) and with diabetic nephropathy (*n* = 382) according to their 24-hour albumin excretion rate (AER) and estimated glomerular filtration rate (eGFR). The patients without diabetic nephropathy who had had at least 5 years of known duration of diabetes had no albuminuria (AER < 30 mg/24 h) and an eGFR > 60 mL min^−1^ 1.73 m^−2^ and were not receiving antihypertension treatment. The patients with diabetic nephropathy had overt albuminuria (AER > 300 mg/24 h) and eGFR < 60 mL min^−1^ 1.73 m^−2^ (no end-stage renal disease or kidney transplantation), without any clinical or laboratory evidence of other kidney diseases. All patients underwent a complete eye examination that included dilated retinal examination and fundus photography or fundus fluorescein angiography. Diabetic retinopathy was evaluated by an experienced ophthalmologist. This study was approved by the ethics committee of the First Affiliated Hospital of Nanchang University, and informed consent was obtained from all subjects.

### 2.2. Biochemical Analysis

All of the patients underwent a standardized clinical and laboratory evaluation. Fasting blood samples were taken for measurement of fasting blood glucose, hemoglobin A1c (HbA1c), total triglyceride (TG), total cholesterol (TC), high density lipoprotein cholesterol (HDL-C), low density lipoprotein cholesterol (LDL-C), and serum creatinine. Fasting blood glucose, TG, TC, HDL-C, LDL-C, and serum creatinine were analyzed using an automated Olympus AU5421 chemistry analyzer (Olympus, Shizuoka, Japan). HbA1c content was measured using a Bio-Rad D-10 glycated hemoglobin analyzer (Bio-Rad, Hercules, USA). Consider eGFR (mL min^−1^ 1.73 m^−2^) = 186 × [serum creatinine (mg/dL)^−1.154^  × age (years)^−0.203^] × (0.742, if female) [12].

### 2.3. Genotyping

For genotype analysis, genomic DNA was extracted from peripheral blood leukocytes of each individual using a DNA isolation kit (Bioteke, Beijing, China). The genomic DNA was subjected to polymerase chain reaction (PCR) with the following primers: Primer-F: 5′-taatttcctgccccattctg-3′ and Primer-R: 5′-catcgcaatctatgcctcct-3′. PCRs were performed in a 10 *μ*L reaction volume containing 150 ng of template DNA, 0.2 *μ*mol/L of forward and reverse primers, 0.225 U* Taq* DNA polymerase, 200 *μ*mol/L of each deoxynucleotide phosphate, and 2.0 mmol/L Mg^2+^ with a ABI 7300 PCR system (Applied Biosystems, Foster City, CA, USA). The amplification protocol consisted of 94°C for 5 min, followed by 35 cycles of denaturation at 94°C for 20 s, annealing at 58°C for 30 s, and extension at 72°C for 30 s, with a final extension at 72°C for 5 min. For restriction fragment-length polymorphism analysis, PCR products were digested with* BsmF1* for 2 h at 65°C. Digestion products were resolved on a 2.5% agarose gel by electrophoresis at 220 V for 30 min. The 2184G minor allele mutation introduces a* BsmF1* restriction site into the gene. Therefore, diagnostic* BsmF1* digestion produced fragments of 160 base pairs (bp) and 236 bp for the mutated minor allele 2184G, while the wild-type major allele 2184A, which does not contain this restriction site, produced a single fragment of 396 bp in length. Twenty-four representative samples from each genotype were further sequenced to confirm the overall genotyping results.

### 2.4. Statistical Analysis

The sample size of this study was established on the basis of our pilot study, which indicated that the genotypic frequency of 2184AG + GG would be 0.21 for the no diabetic nephropathy group and 0.13 for the diabetic nephropathy group. It was calculated that the number of subjects needed to achieve 80% power to detect a difference between the two groups, with a significance level *α* (chance of a two-sided *α* error) of 0.05, was 342. In our study, 486 patients with no diabetic nephropathy and 382 patients with diabetic nephropathy were enrolled. Therefore, the sample size was considered to be adequate.

All statistical analyses were performed using SPSS 17.0 (SPSS Inc. Chicago, IL, USA) for Windows. The clinical and laboratory continuous variables are expressed as means ± standard deviation (SD) or as medians (interquartile range) if the distribution of the variable was found to be nonnormally distributed. Comparisons of the clinical and laboratory continuous variables between the diabetes groups with and without diabetic nephropathy as well as those between the genotypic groups were performed with unpaired Student's *t*-tests. Nonnormally distributed variables were logarithmically transformed before analysis. Categorical variables and Hardy-Weinberg equilibrium were analyzed by an *χ* ^2^ test. Intergroup comparisons of genotype distribution and allele distribution were analyzed by Fisher's exact test or an *χ* ^2^ test, as appropriate. To evaluate the independent contribution of the 2184 polymorphism to the risk of diabetic nephropathy, we performed multivariate logistic regression analysis of type 2 diabetes patients with diabetic nephropathy and controls with type 2 diabetes without diabetic nephropathy. The analyses included possible confounders such as sex, age at diagnosis of diabetes, BMI, smoking, diabetes known duration, hypertension, TG level, TC level, LDL-C level, HDL-C level, and HbA1C. Odds ratios (ORs) and 95% CIs were calculated. Power of sample size for the single nucleotide polymorphism at 5% significance level and 80% power was calculated by genetic power calculator according to our pilot studies, and sample size was kept accordingly. A *P* value <0.05 was considered statistically significant.

## 3. Results

### 3.1. Clinical and Laboratory Characteristics of Patients with Type 2 Diabetes with and without Diabetic Nephropathy

As shown in Table 1, there were significant differences in age of onset, known duration of diabetes, hypertension, systolic blood pressure, diastolic blood pressure, LDL-C, retinopathy, and hypoglycemic treatments between type 2 diabetes patients with and without diabetic nephropathy.

### 3.2. Clinical and Laboratory Characteristics of Patients with Type 2 Diabetes Classified according to Their 2184A/G Genotypes

Because the frequency of the GG genotype was low, we divided the enrolled subjects into two groups: AA and AG + GG. As shown in Table 2, the levels of AER and serum creatinine were significantly lower and *C*_cr_ was significantly higher in patients with the AG + GG genotype compared with the patients with the AA genotype. There were no significant differences in other clinical and laboratory characteristics between the AG + GG and AA genotype groups.

### 3.3. Association of 2184A/G Polymorphism with Diabetic Nephropathy in Patients with Type 2 Diabetes

As shown in Table 3, the genotypic distribution of the 2184A/G polymorphism in each group attained Hardy-Weinberg equilibrium (*P* > 0.05). The frequency of the AA, GA, and GG genotypes was 79.6, 19.4, and 1.0%, respectively, in the group without diabetic nephropathy, compared with 86.9, 12.8, and 0.3%, respectively, in the group with diabetic nephropathy. The allelic frequencies of the A and G alleles were 88.1 and 11.9% in the no diabetic nephropathy group versus 93.1 and 6.9% in the diabetic nephropathy group. Because the frequency of the GG genotype was low, we divided the enrolled subjects into two groups, AA and AG + GG. The AG + GG genotype frequency was significantly lower in the diabetic nephropathy group than in the no diabetic nephropathy group (*χ* ^2^ = 7.97, *P* = 0.005). The G allele frequency was also significantly lower in the diabetic nephropathy group than in the no diabetic nephropathy group (*χ* ^2^ = 11.91, *P* = 0.001). After adjustments for possible confounders, multivariate logistic regression analyses showed that the 2184A/G polymorphism was independently associated with diabetic nephropathy (OR = 0.46, 95% CI: 0.22–0.92, *P* = 0.028).

## 4. Discussion

This study found that the 2184A/G polymorphism in the RAGE gene was significantly associated with overt diabetic nephropathy in Chinese patients with type 2 diabetes, even after adjustments for possible confounders. We observed fewer patients with diabetic nephropathy with the GG + AG genotype of the 2184A/G polymorphism than in those with the AA genotype.

These results indicate that the GG + AG genotype may have a protective role against diabetic nephropathy. However, our results differ from the results of the study by Kaňková et al. [13], which showed that patients with type 2 diabetes from central Europe with the GG + AG genotype of the 2184A/G polymorphism are at risk of diabetic nephropathy. They also found that the 2245G/A polymorphism located at intron 8 of the RAGE gene, similar to 2184A/G polymorphism, may have a protective role against diabetic nephropathy, which is inconsistent with the study by Ng et al. [14]. Because diabetic nephropathy and diabetic retinopathy are diabetic microvascular complication, they have some similar pathogenesis.

The different genetic and environmental contributions of disease risk in various ethnic and geographic groups might explain the contrary results. In patients with type 2 diabetes without diabetic nephropathy, the 2184G allele frequency in the Chinese population (9.0%) in the present study was lower than that in the central European population (19.1%) [15], and in type 2 diabetes patients with diabetic nephropathy, the 2184G allele frequency (6.9%) in the Chinese population in our study was considerably lower than that in the central European population (23.0%) [13]. Moreover, there are ethnic heterogeneities in other polymorphisms in the RAGE gene, such as −429T/C, −374T/A, and Gy82Ser, between the Chinese and other populations [16, 17]. In our previous study, the −429C allele frequency (12.7%) of −429T/C polymorphism and the −374A allele frequency (13.9%) of −374T/A polymorphism in Chinese Han were lower than those in Caucasians (18% and 20%, resp.) [17, 18].

Different results have been reported in case-control studies on the relationship between these polymorphisms and diabetic retinopathy and angiopathy in various ethnic populations. Ng et al. [19] reported that the −429T/C polymorphism is associated with an increased risk for diabetic retinopathy in patients with type 2 diabetes. In contrast, the study by Ng et al. [14] and our previous studies [17, 19] showed that the two polymorphisms are not associated with diabetic retinopathy in Chinese and Malaysian patients with type 2 diabetes. There are also inconsistent results with the relationship between these polymorphisms and diabetic angiopathy in meta-analysis studies [20, 21]. Additionally, the type and duration of diabetes and other characteristics, such as the patients who were selected and the sample size, might vary between the studies and, thus, have influenced the results.

The cellular receptor, RAGE, with multiple ligands including AGEs, high mobility group box 1, and S100/calgranulins, is expressed in a wide variety of cell types such as endothelial cells, smooth muscle cells, phagocytes, and neurons [8]. Interactions between RAGE and AGEs activate multiple signaling pathways and subsequently evoke oxidative stress and inflammatory responses in vascular cells, which lead to the development and progression of diabetic nephropathy [9]. The circulating soluble RAGE form (sRAGE) prevents binding of RAGE ligands to cellular RAGE and thereby serves as a decoy for ligands in preventing these RAGE ligand interactions [22]. At least two isoforms of sRAGE are known, a cleaved isoform caused by cleavage of the receptor from the cell membrane and a spliced isoform (esRAGE) arising from alternative splicing of mRNA, which involves the regions between introns 7 and 9 [23, 24]. The 2184A/G polymorphism, which is located on intron 8 of the RAGE gene, has been postulated to be involved in the regulation of sRAGE production. Kalousová et al. [25] reported that the −429T/C and 2184A/G polymorphisms are associated with circulating sRAGE level in chronic hemodialysis patients, and the highest sRAGE levels are found in the −429 CC and 2184 GG genotypes of the RAGE gene. These results support the observations of the present study.

The initial evidence of diabetic nephropathy in patients with type 2 diabetes is the development of micro- and macroalbuminuria. However, there is considerable evidence to suggest that patients with type 2 diabetes can commonly progress to a significant degree of renal impairment while remaining normoalbuminuric [1]. The UK Prospective Diabetes Study [26] demonstrated that 51% of patients who progress to chronic renal failure had no preceding albuminuria. MacIsaac et al. [27] reported that 23% of patients with type 2 diabetes who have impaired renal function (eGFR < 60 mL·min^−1^·1.73 m^−2^) have normoalbuminuria after accounting for the use of RAS inhibitors. Although normoalbuminuria in type 2 diabetes patients with impaired renal function raises the possibility of nondiabetic chronic kidney disease, many of these patients have diabetic nephropathy, especially in the presence of retinopathy [28]. Therefore, a portion of patients with diabetic nephropathy may be missed if diagnosis only depends upon increased albuminuria [29]. In the present study, identification of diabetic nephropathy depended upon AER and eGFR screening. In patients with normoalbuminuria and eGFR < 60 mL·min^−1^·1.73 m^−2^, those with retinopathy were included in the diabetic nephropathy group, and those without retinopathy were excluded.

The present study has some limitations. First, we did not measure the serum levels of sRAGE and, therefore, did not judge whether the serum levels of sRAGE were concomitantly associated with the 2184A/G polymorphism of the RAGE gene and diabetic nephropathy. Second, this was a cross-sectional study, which can only demonstrate associations and not causal relationships between the 2184A/G polymorphism and diabetic nephropathy. Although a sufficient sample size was provided and the power values of the analysis were more than 90%, further studies in a prospective cohort with a large population are needed to fully elucidate the relationship between the 2184A/G polymorphism and diabetic nephropathy.

## 5. Conclusions

In conclusion, the present study indicated that the 2184A/G polymorphism in the RAGE gene was significantly associated with diabetic nephropathy in Chinese Han patients with type 2 diabetes.

## Figures and Tables

**Table 1:** Clinical and laboratory characteristics of patients with type 2 diabetes with and without diabetic nephropathy.

	Without diabetic nephropathy	With diabetic nephropathy	*P* ^*∗*^
*n*	486	382	—
Sex (male/female)	248/238	217/165	0.09
Age (years)	60.8 ± 11.7	61.6 ± 11.0	0.101
Age at diagnosis of diabetes (years)	51.2 ± 12.2	53.1 ± 11.8	0.030
Known duration of diabetes (years)	8.2 (5.6–12.1)	9.3 (3.7–13.6)	0.019
Smoking (%)	56 (11.5)	49 (12.8)	0.558
Body mass index (kg/m^2^)	23.7 ± 3.2	23.4 ± 3.0	0.756
Hypertension (%)	144 (29.6)	225 (58.9)	<0.001
Systolic blood pressure (mmHg)	131.0 ± 17.5	141.4 ± 21.0	<0.001
Diastolic blood pressure (mmHg)	80.9 ± 11.3	82.9 ± 11.9	0.035
HbA1c (%)	8.58 ± 2.26	8.51 ± 2.24	0.726
Fasting blood glucose (mmol/L)	8.61 ± 3.32	8.59 ± 2.89	0.653
Total cholesterol (mmol/L)	4.47 ± 1.31	4.45 ± 1.29	0.695
Triglycerides (mmol/L)	1.36 (0.84–1.93)	1.39 (0.87–2.16)	0.630
LDL-cholesterol (mmol/L)	3.06 ± 0.79	3.69 ± 0.81	0.009
HDL-cholesterol (mmol/L)	1.15 (0.89–1.32)	1.10 (0.83–1.29)	0.639
Retinopathy (%)	100 (20.6)	244 (63.7)	<0.001
Hypoglycemic treatments			
OHA (%)	185 (38.1)	101 (26.4)	<0.001
Insulin (%)	130 (26.7)	165 (43.2)	
Insulin + OHA (%)	171 (35.2)	116 (30.4)	

Data are means ± SD, medians (interquartile range), or *n* (%). ^*∗*^ *P* values were obtained by an unpaired Student's *t*-test analysis or *χ* ^2^ test, as appropriate. OHA, oral hyperglycemic agent.

**Table 2:** Clinical and laboratory characteristics of patients with type 2 diabetes classified according to their 2184A/G genotypes.

	AA	AG + GG	*P* ^*∗*^
*n*	719	149	—
Sex (male/female)	379/340	86/63	0.265
Age (years)	61.1 ± 11.3	61.3 ± 11.8	0.569
Age at diagnosis of diabetes (years)	52.6 ± 12.5	52.6 ± 12.1	0.894
Known duration of diabetes (years)	9.1 (4.0–13.2)	9.0 (3.9–14.1)	0.247
Smoking (%)	87 (12.1)	18 (12.1)	0.995
Body mass index (kg/m^2^)	23.8 ± 3.5	23.2 ± 3.7	0.084
Hypertension (%)	308 (42.8)	61 (40.9)	0.670
Systolic blood pressure (mmHg)	136.7 ± 16.8	133.2 ± 15.3	0.122
Diastolic blood pressure (mmHg)	81.5 ± 11.6	80.8 ± 10.5	0.216
HbA1c (%)	8.79 ± 2.27	8.66 ± 2.31	0.108
Fasting blood glucose (mmol/L)	8.52 ± 3.05	8.59 ± 3.86	0.769
Total cholesterol (mmol/L)	4.52 ± 1.37	4.39 ± 1.32	0.099
Triglycerides (mmol/L)	1.40 (0.88–2.32)	1.37 (0.83–2.17)	0.113
LDL-cholesterol (mmol/L)	3.58 ± 0.84	3.41 ± 0.79	0.091
HDL-cholesterol (mmol/L)	1.12 (0.80–1.25)	1.14 (0.81–1.28)	0.236
Serum creatinine (*μ*mol/l)	70.0 (46.0–98.6)	56.6 (41.3–83.5)	0.031
eGFR (mL·min^−1^·1.73 m^−2^)	109 (86.5–137.4)	37.6 (23.8–51.7)	<0.001
AER (mg/24 h)	46.4 (9.8–127.5)	31.2 (6.9–110.4)	0.013
Retinopathy (%)	291 (40.5)	53 (35.6)	0.266
Hypoglycemic treatments			
OHA (%)	232 (32.3)	54 (36.2)	0.575
Insulin (%)	249 (34.6)	46 (30.9)	
Insulin + OHA (%)	238 (33.1)	49 (32.9)	

Data are means ± SD, medians (interquartile range), or *n* (%). ^*∗*^ *P* values were obtained by an unpaired Student's *t*-test analysis or *χ* ^2^ test, as appropriate. OHA, oral hyperglycemic agent.

**Table 3:** The genotypic and allelic distribution of the 2184A/G polymorphism in in type 2 diabetic patients with and without diabetic nephropathy.

	Without diabetic nephropathy *n* (%)	With diabetic nephropathy *n* (%)	*χ* ^2^	*P* ^*∗*^
Genotype				
AA	387 (79.6)	332 (86.9)	7.97	0.005
AG + GG	99 (20.4)	50 (13.1)		
Allele				
A	768 (88.1)	693 (93.1)	11.91	0.001
G	104 (11.9)	51 (6.9)		

^*∗*^ *P* values were obtained by an *χ* ^2^ test.
